# Prescribing cycle training intensity from the six-minute walk test for patients with COPD

**DOI:** 10.1186/1471-2466-7-9

**Published:** 2007-07-01

**Authors:** Muhammad R Zainuldin, Danielle Knoke, Martin G Mackey, Nia Luxton, Jennifer A Alison

**Affiliations:** 1 Discipline of Physiotherapy, The University of Sydney, Sydney, Australia; 2 Department of Physiotherapy, Royal Prince Alfred Hospital, Sydney, Australia

## Abstract

**Background:**

Cycle training intensity for patients with chronic obstructive pulmonary disease (COPD) is normally based on an incremental cycle test. Such tests are expensive and not readily available to clinicians. The six-minute walk test (6MWT) has been proposed as an alternative to an incremental cycle test for this purpose, based on the findings of previous research that the peak oxygen consumption (VO_2peak_) for the incremental cycle test and the 6MWT was equivalent in participants with COPD. A regression equation relating distance walked on the 6MWT and peak work rate (W_peak_) on the incremental cycle test has been described. The aim of this study is to measure the physiological responses to constant load cycle exercise performed at an intensity of 60% W_peak _determined from the 6MWT in participants with stable COPD.

**Methods/Design:**

This study is a prospective, repeated measures design. Thirty-five participants with stable COPD and mild to severe lung disease will be recruited from referrals to pulmonary rehabilitation. Subjects with co-morbidities limiting exercise performance will be excluded. Two 6MWTs will be performed. The better 6MWT will be used to calculate W_peak _for cycle exercise from a regression equation. After 30 minutes rest, subjects will perform ten minutes of constant-load cycle exercise at 60% of the calculated W_peak_. During all exercise, cardiorespiratory and metabolic data (Cosmed K4b^2^), dyspnoea and rate of perceived exertion (RPE) will be recorded. The VO_2 _measured at the end of cycle exercise will be compared to VO_2peak _of the 6MWT (VO_2bike_/VO_2walk_). Pearson's correlation coefficient will be calculated for the relationship between VO_2bike _and VO_2walk_. A one-way analysis of variance (ANOVA), with Bonferroni correction, will be performed to determine whether the ratio of VO_2bike_/VO_2walk _is affected by disease severity.

**Discussion:**

This novel study will measure the physiological responses to cycle exercise, in terms of VO_2peak_, performed at an intensity determined from the 6MWT in participants with COPD. Positive findings will enable clinicians to more precisely prescribe cycle training intensity by utilising a simple, reliable and inexpensive 6MWT, thus providing a better standard of care for patients with COPD referred to pulmonary rehabilitation.

**Trial Registration:**

ACTRNO12606000496516

## Background

Exercise training has been regarded as the cornerstone of pulmonary rehabilitation for patients with chronic obstructive pulmonary disease (COPD) [1] and highly recommended as efficacious treatment in reducing disability [2]. An appropriate prescription of exercise for patients with COPD is important in ensuring that an endurance training program in pulmonary rehabilitation results in peripheral adaptations, reduced dyspnoea and fatigue in daily activities, and improved function [1,3-5]. Constant-load submaximal cycle exercise is one of the most common modes of endurance training in pulmonary rehabilitation [6], and has been shown to result in peripheral adaptations [4] and increased exercise capacity [3,4]. In order to prescribe exercise, each patient's exercise capacity needs to be assessed [7]. A maximal incremental cycle ergometer test is the recommended assessment protocol to obtain peak oxygen consumption (VO_2peak_), and is regarded to be the gold standard of exercise testing for patients with COPD [7,8]. The intensity for cycle exercise training is normally prescribed from a percent of VO_2peak _or peak work rate (W_peak_) obtained from an incremental cycle test [7].

The incremental cycle test is most commonly performed in a laboratory and requires sophisticated equipment and well-trained staff [7,8]. However, such testing for the large number of patients referred to pulmonary rehabilitation may not be feasible due to cost and lack of resources. The ability to formulate an appropriate prescription of exercise may be compromised if such an exercise test cannot be performed.

An alternative test, a six-minute walk test, has been proposed as a basis for exercise prescription [9]. The six-minute walk test (6MWT) is less costly than the incremental cycle test because the walking test does not require sophisticated equipment and technical training [10]. Previous studies have shown that the 6MWT produced similar VO_2peak _as the incremental cycle test in participants with COPD, validating the capability of the 6MWT to assess exercise capacity in this population [9,11]. In addition, the six-minute walk distance (6MWD) is significantly correlated with both VO_2peak _[10,12] and W_peak _on the incremental cycle test in patients with COPD [9]. From the correlation found between 6MWD and work rate on the incremental cycle test, Luxton et al (2005) proposed that a cycle training intensity could be calculated from W_peak _in COPD [9]. It is important to determine whether this method of calculating cycle training intensity produces adequate physiological responses (in terms of VO_2peak_) that would result in a training effect in patients with COPD. There have been no reports of cycle training exercise prescription from the 6MWT.

An intensity that exceeds 50% VO_2peak _is regarded as sufficient to elicit training effects in subjects with COPD [13], however, an upper limit of % VO_2peak _for cycle training intensity has not been reported [6]. In terms of peak work rate, the recommended training intensity range is from 50% to 80% of W_peak _in COPD [3].

The aim of this study is to measure the physiological responses to cycle exercise performed at an intensity determined from the 6MWT in participants with COPD. It is hypothesised that cycle exercise at 60% W_peak_, calculated from the results of the 6MWT, will produce a VO_2 _above 50% VO_2peak _when compared with peak VO_2 _from the 6MWT.

## Method/Design

### Participants

Participants with diagnosed stable COPD, as defined by the COPDX Guidelines [14], will be recruited for this study through advertisement or personal contact with patients referred to the pulmonary rehabilitation unit at Royal Prince Alfred Hospital, Sydney, Australia. Participants with mild to severe lung disease will be included. Mild lung disease is characterised by forced expiratory volume in one second (FEV_1_) of 60 to 80% predicted, moderate by FEV_1 _of 40% to 59% predicted and severe by FEV_1 _of less than 40% predicted [14]. The study has been approved by the Human Research Ethics Committees of The University of Sydney and Royal Prince Alfred Hospital. Prior to testing, written, informed consent will be obtained from all participants.

Participants will be excluded if they present with a fever or increased sputum; a hospital admission in the previous month; are unable to wear a facemask due to claustrophobia or are on long term oxygen therapy. Participants with cardiovascular, neurological and musculoskeletal co-morbidities that may limit exercise performance will also be excluded [11,12].

### Sample size calculation

Thirty-five participants will be required based on a calculation of a 95% confidence interval of 0.65 – 0.95 for the predicted relationship of VO_2peak _between the 6MWT and the incremental cycle test to achieve a power of 0.80. We will enrol 39 participants to allow for 10% drop-out.

### Procedures

**Figure 1:**
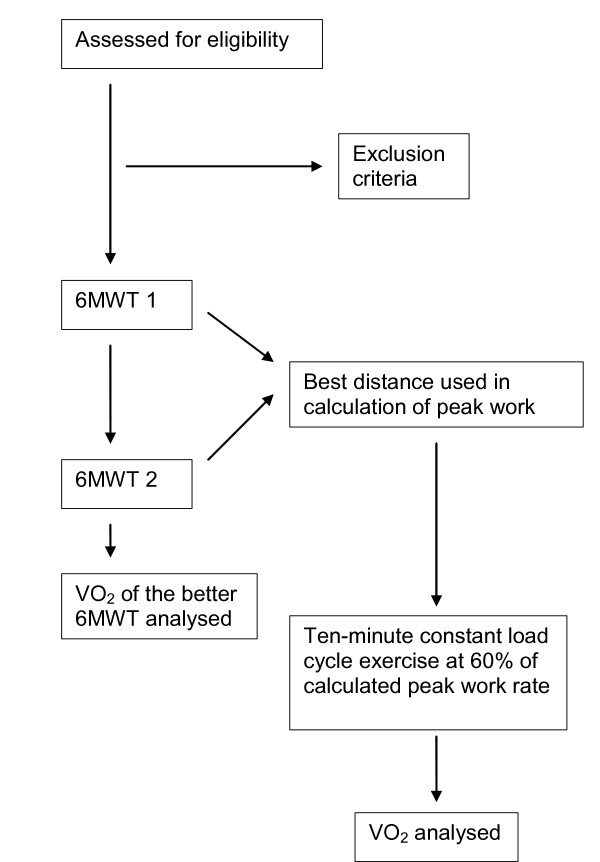
Research design.

This study will use a prospective, repeated measures design (Figure 1). All participants will perform two 6MWTs and a ten-minute constant-load cycle exercise in that order. The 6MWT will be performed prior to the cycle exercise as the results of the 6MWT will be required to calculate the intensity of cycle exercise. Participants will be requested to avoid having caffeinated drinks, a heavy meal or exercising for at least two hours before the tests [7,8]. All testing will be performed on one occasion. A minimum of thirty minutes' rest will be given between the two walking tests and again before the cycle exercise, in order to allow for physiological parameters to return to baseline levels. Spirometry (Niche EasyOne hand-held spirometer, Device Medical Technologies, Zurich, Switzerland), including measures of FEV_1_; FEV_1 _percent predicted (FEV_1 _%pred); forced vital capacity (FVC); and FVC percent predicted (FVC %pred), will be performed prior to the walking and cycling tests [15]. Participants will be asked to take their prescribed bronchodilators prior to testing.

The 6MWTs will be performed on a 32-metre continuous rectangular track with standardised instructions and encouragement given at every minute [10,12]. A second 6MWT will be performed by all participants to account for a learning effect [10,16]. The longer 6MWD will be entered into a regression equation determined from a previous study [9 and personal communication] to calculate predicted peak watts on a cycle ergometer. Subsequently, 60% of the predicted peak work rate will be prescribed for the constant-load cycle exercise [4]. In this study, 60% W_peak _has been chosen from the recommended range of 50% to 80% because it has been shown that this training intensity is achievable and produced physiological training adaptations for patients with moderate to severe COPD [4].

The cycle exercise will be performed on an electronically braked cycle ergometer (Ergomed EM 840, Siemens Medical Engineering Group, Erlangen, Germany). Participants will pedal between 50–60 rpm throughout the exercise [7]. The intensity will be steadily ramped from unloaded to the designated load over two minutes, according to the American Thoracic Society and American College of Chest Physicians guidelines [7]. Subsequently, participants will pedal at a constant load for a further ten minutes. This duration of exercise was selected because it is sufficient to attain steady-state responses [17] and it is similar to an initial training session as patients with COPD are usually unable to exercise continuously for more than ten minutes at the start of pulmonary rehabilitation [17]. The test will be terminated if the participants suffer severe dyspnoea or fatigue or if oxygen saturation (SpO_2_) falls below 85%.

### Measurements

Breath-by-breath values for oxygen consumption (VO_2_), carbon dioxide production (VCO_2_), minute ventilation (V_E_), tidal volume (V_T_), frequency of breathing (fb) and respiratory quotient (R) will be measured during each test using a portable metabolic system (Cosmed K4b^2^, Rome, Italy). The participants will wear a small battery pack and portable gas analyser, weighing less than a kilogram, on their chests and will breathe through a flexible rubber facemask with the flowmeter attached to the system [18]. Heart rate (HR) and SpO_2 _will be measured using a portable heart rate monitor (Polar ^®^, Polar Electro, Oy, Finland) and an adult articulated finger clip sensor (Model 8000AA, Nonin Medical Inc, Minnesota, USA), respectively. Data are either transmitted to a Windows-based computer by telemetry using the Cosmed patented software, or stored in its memory unit. The portable metabolic system has been previously utilised to measure responses during 6MWT in patients with COPD [9,18,19].

All physiological values will be averaged for the last twenty seconds of each completed minute and twenty seconds prior to the end of each exercise test. The modified Borg Category Ratio 0–10 scale will be used to measure dyspnoea and rate of perceived exertion (RPE) at the beginning, and during each minute of cycle exercise or at the third minute of 6MWT, at the end of each test, and also after a minute of recovery [20].

Maximal voluntary ventilation (MVV) will be calculated from the equation, FEV_1 _× 35.5 [21]. V_E _at the end of the constant-load cycle exercise will be compared to MVV, i.e. the ratio V_E_/MVV is expressed as a percentage to represent ventilatory reserve of each participant [7].

Based on earlier findings that peak VO_2 _on the 6MWT and the incremental cycle test were not significantly different [9,11], we will assume that each participant's peak VO_2 _on the 6MWT (VO_2walk_) will be equivalent to their VO_2peak _on an incremental cycle test. In order to determine the exercise intensity during constant-load cycle exercise, the VO_2 _measured from the cycle exercise (VO_2bike_) will be calculated as a percentage of VO_2walk_, i.e. VO_2bike_/VO_2walk _(%). In our study, VO_2bike _will represent the values of VO_2 _averaged for the last 20 seconds of the final three minutes of the cycle exercise. VO_2walk _will represent the peak VO_2 _on the 6MWT calculated from the mean of the last 20 seconds of the final minute.

### Statistical methods

Results will be expressed in mean ± standard deviation (SD). Pearson correlation coefficients will be calculated for the relationship between VO_2bike _and VO_2walk_.

Correlation coefficients will also be calculated for the relationships between VO_2bike_/VO_2walk _and the following parameters: a) body mass index; b) measures of lung function (FEV_1_, FEV_1_%pred, FVC, FEV_1_/FVC ratio); c) 6MWD; d) percentage of predicted 6MWD; e) dyspnoea; and f) RPE scores. These parameters will be investigated to determine their influence on the VO_2 _response to the prescribed cycle exercise intensity. A one-way analysis of variance (ANOVA) with a confidence interval (CI) of 0.95 will be performed in a post-hoc analysis with Bonferroni corrections to determine the effect of disease severity (mild, moderate and severe) on VO_2bike_/VO_2walk_, dyspnoea and RPE during cycle exercise. The level of significance will be set at *p *< 0.05.

Statistical analyses will be performed on SPSS-Windows (release 14: SPSS: Chicago, Illinois, USA).

## Discussion

The significance of the study pertains to the ability of clinicians to prescribe an appropriate level of cycle exercise for patients with COPD referred to pulmonary rehabilitation. If the intensity of cycle training at 60% W_peak_, calculated from the results of the 6MWT, produces a VO_2 _above 50% VO_2peak _when compared with peak VO_2 _from the 6MWT, this simple and inexpensive method of cycle exercise prescription could be adopted by pulmonary rehabilitation centers or community health services in both metropolitan and rural settings. This study will also demonstrate if the exercise intensity prescribed is achievable by patients with varying levels of disease severity. If the study findings show that the prescribed intensity is too high or too low for participants with certain levels of disease severity, a titration of this intensity and/or a revision of the prediction equation may be needed. Although we recognise that it is difficult to define optimal training intensity, this study may bring clinicians closer to the definition by demonstrating the response, in terms of %VO_2peak_, which an exercise intensity of 60% W_peak _elicits. Similarly, patients with COPD referred to pulmonary rehabilitation will benefit from an individualised cycle training program that should be more effective in producing training outcomes, thus enhancing the standard of care. To our knowledge, the proposed research is the first study internationally that will provide a quantitative method for more precise, individualised prescription of cycle training intensity from a simple and inexpensive 6MWT in patients with COPD.

## Competing interests

The author(s) declare that they have no competing interests.

## Authors' contributions

JA had the original idea for the study. JA, MM and NL designed the study. MZ and DK contributed to refining the study design. MZ drafted the manuscript. All authors revised the manuscript and have approved the final version.

## Pre-publication history

The pre-publication history for this paper can be accessed here:


